# Access to and Quality of Healthcare for Pregnant and Postpartum Women During the COVID-19 Pandemic

**DOI:** 10.3389/fgwh.2021.628625

**Published:** 2021-02-10

**Authors:** Áine Brislane, Fionnuala Larkin, Helen Jones, Margie H. Davenport

**Affiliations:** ^1^School of Science, Technology and Health, York St. John University, York, United Kingdom; ^2^School of Education, Language and Psychology, York St. John University, York, United Kingdom; ^3^Research Institute of Sport and Exercise Sciences, Liverpool John Moores University, Liverpool, United Kingdom; ^4^Program for Pregnancy and Postpartum Health, Faculty of Kinesiology, Sport, and Recreation, Women and Children's Health Research Institute, Alberta Diabetes Institute, University of Alberta, Edmonton, AB, Canada

**Keywords:** COVID-19, obstetric care, pregnancy, postpartum, quality of care

## Abstract

**Introduction:** During the COVID-19 pandemic, obstetric care has adopted new precautions to ensure services can be maintained for pregnant women. The aim of this study was to describe access to and quality of obstetric care for pregnant and postpartum women during the COVID-19 pandemic and to identify factors that predict quality of care at this time.

**Methods:** Between May 3 and June 28, 2020, we recruited women who were pregnant or within the first 6 months after delivery to participate in an online survey. This included questions on access to obstetric healthcare (type and place of health care provider, changes to obstetric appointments/services, appointment preferences) and the Quality of Prenatal Care Questionnaire (QPCQ).

**Results:** Of the 917 eligible women, 612 (67%) were pregnant and 305 (33%) were in the first 6 months after delivery. Sixty-two percent (*n* = 571) reported that COVID-19 had affected their healthcare; appointments were rearranged, canceled or occurred *via* virtual means for 29% (*n* = 166), 29% (*n* = 167), and 31% (*n* = 175) of women, respectively. The majority preferred to physically attend appointments (74%; *n* = 676) and perceived the accompaniment of birth partners as important (77%; *n* = 471). Sixty-two percent (*n* = 380) were permitted a birth partner at delivery, 18% (*n* = 111) were unsure of the rules while 4% (*n* = 26) were not permitted accompaniment. During pregnancy, QPCQ was negatively associated with disruption to obstetric services including exclusion or uncertainty regarding birth partner permissions [*F*_(7, 433)_ = 11.5, *p* < 0.001, *R*^2^ = 0.16] while QPCQ was negatively associated with inadequate breastfeeding support postpartum [*F*_(1, 147)_ = 12.05, *p* = 0.001, *R*^2^ = 0.08].

**Conclusion:** Pregnant and postpartum have experienced disruption in their access to obstetric healthcare. Perceived quality of obstetric care was negatively influenced by cancellation of appointment(s), suspension of services and exclusion of birth partners at delivery. During this time, continuity of care can be fulfilled *via* virtual and/or phone appointments and women should receive clear guidance on changes to services including birth partner permissions to attend delivery.

## Introduction

In January 2020, the World Health Organization declared a public health emergency in response to the rising incidence of the 2019 novel coronavirus (COVID-19) that was later declared a pandemic in March 2020. In the early stages of the pandemic, pregnant women were categorized as high-risk and advised to limit social interactions to protect themselves against contracting the virus. As a result, clinical care has adopted new precautions to ensure that services can be maintained for pregnant women. In the US, these precautions include the use of personal protective equipment, physical distancing, frequent hand washing, and limiting contact with others (1). This is similar in the UK alongside permission for asymptomatic partners to attend births (2). Despite this, emerging evidence indicates that for many women, services are being disrupted and include suspended and/or canceled appointments, restrictions regarding place of birth, continuity of care (3), and much ambiguity regarding birth partner permissions to attend delivery (4, 5). Disrupted access to healthcare appears to be a global consequence of the COVID-19 pandemic (6–11). This is of particular concern with respect to obstetric practice because limited access to services increase the risks of adverse health outcomes for both mother and baby (12).

In an effort to protect against COVID-19 transmission, many pregnant and postpartum women are fulfilling appointments by telephone and video teleconferencing. While this adaptation to continuity of care is extremely encouraging, it is plausible that reduced face-to-face interaction may invoke a perception of limited healthcare access among pregnant and postpartum women (13). In an effort to combat this plausible perception, women are encouraged to avail of information online regarding their pregnancy and associated COVID-19 risks (14). Women are also encouraged to engage with support groups to limit pandemic-related feelings of isolation (13, 15) that can have adverse outcomes for mother and baby (16). To ensure that such online resources and support groups are effective in benefitting women during this time, it is paramount to know the type and format of information women would like to receive.

Pandemic associated disruptions in accessing healthcare has negative consequences for quality of care (17, 18). According to Heaman et al. (19), prenatal quality of care is underpinned by constructs that include information sharing, anticipatory guidance, sufficiency, approachability, and availability. During the COVID-19 pandemic, it is plausible that these constructs are perturbed given the need for the maternity environment to rapidly adapt (3). For example, during the pandemic, obstetric caregivers are tasked with staying informed and adapting to guidance regarding the availability of services and the impact of COVID-19 on pregnant women and their babies (20). As the guidance emerges and evolves, it may not always be possible for caregivers to provide this information. Furthermore, the redeployment of midwives to general nursing roles, reductions in staff numbers due to COVID-19 related sickness, the implementation of virtual instead of face-to face appointments (3), restrictions on both home births (21), and community visits (22) may negatively impact the sufficiency of services, the approachability, and availability of staff. As a result, quality of care for pregnant and postpartum women may be directly impacted by the COVID-19 pandemic however this remains to be determined.

The primary aim of this study was to describe access to (e.g., appointment fulfillment, cancellations, virtual means, and service suspensions) and quality of obstetric care (e.g., information sharing, anticipatory guidance, sufficiency, availability, and approachability) for pregnant and postpartum women during the COVID-19 pandemic. A secondary aim was to identify factors that predict quality of care in pregnant and postpartum women. Lastly, we aimed to explore what information would benefit pregnant and postpartum women during a pandemic to help inform clinical and research practice. The study was approved by the Ethics Committee at York St. John University (Reference number: STHEC0011) and adhered to the ethical statements outlined in the Declaration of Helsinki apart from registry in a publicly accessible database.

## Methods

### Sample Design

Between May 3 and June 28, 2020, women that self-reported as pregnant or in the first 6 months following delivery completed an online questionnaire advertised *via* social media platforms (Facebook, Twitter, Reddit) and shared publicly to facilitate snowball sampling. Women were ineligible to participate if not currently pregnant, or not within the first 6 months following delivery. Participants were made aware of the study aims, risks, and benefits alongside reassurance of freedom to withdraw from the questionnaire at any time-point. Electronic consent was requested before progressing to the survey.

### Variables Assessed

Participants answered questions on demographic factors including their age, level of education, ethnicity, employment status, health, and reproductive history. They responded to questions about symptoms, testing, and diagnosis of COVID-19 they experienced during/following pregnancy. The authors liaised with a midwife to confirm aspects of healthcare access to be captured in the questionnaire. Participants were asked about their current level of access to obstetric healthcare, including (1) the type of health care provider (e.g., obstetrician, midwife, general practitioner, a combination of each), (2) the place at which they received their care (hospital, family practice, private clinic, or other), (3) any changes in obstetric appointments or services (e.g., unchanged, canceled, or modified schedule; ability of their partner to attend appointments; transportation to appointments), and (4) any appointment preferences they had (physical attendance, virtual, home visit, no appointment). Pregnant women were asked about the impact of COVID-19 on their birth plans including birth partner permissions to attend and their feelings about this. All women were asked an open-ended question about what type of pregnancy related information would be/had been useful for them during this time (i.e., during a global pandemic).

#### Quality of Prenatal Care Questionnaire

All participants completed a validated questionnaire to quantify quality of healthcare using the 46-item Quality of Prenatal Care Questionnaire (QPCQ) (19). While the questionnaire is predominantly intended for use during pregnancy, it has been deemed a valid and reliable instrument to assess the relationship between quality of care and maternal health outcomes (23–25). Pregnant and postpartum women were asked to complete the questionnaire with their most recent pregnancy related appointment in mind.

The QPCQ is a self-report instrument that quantifies quality of prenatal care using a 5-point Likert scale ranging from 1 (strongly disagree) to 5 (strongly agree). It is comprised of six subscales that include information sharing (9 items), anticipatory guidance (11 items), sufficient time (5 items), approachability (4 items), availability (5 items), and support and respect (12 items). The sum of the QPCQ subscales are calculated and presented as a total score ranging from 46 to 230 with higher values indicating better quality of care. The total score obtained is divided by 46, and the score of each subscale is divided by the respective number of questions within that subscale. The mean score obtained (total or subscale) can range from 1 to 5, again with the higher value representative of better care quality. Cronbach's alpha was applied to each of the six subscales (α = 0.91, 0.92, 0.92, 0.87, 0.89, and 0.97, respectively) and totaled score of the QPCQ for internal consistency (α = 0.97). To contextualize participant QPCQ responses, the total score was expressed as a percentage of the possible maximum score (230), with scores at or over 70% indicating participant care was *good*, and scores under 70% indicating that care was *poor* (26). This percent score and dichotomous coding was used in subsequent statistical analyses.

#### Thematic Coding

The open-ended questions were analyzed by a researcher with an undergraduate degree in Psychology, who was blind to the study hypothesis and all other data on participants. The questions coded included, although were not limited to, items such as “How does [your partner being permitted/not permitted to attend the birth] make you feel?” A qualitative content analysis was conducted on six open ended questions separately in accordance with relevant guidelines (27). The researcher immersed themselves in the responses and devised a categorical coding scheme for each question to reflect emerging themes from the responses (e.g., “anxious,” “relieved,” “sad,” “alone”) to enable subsequent input into a statistical model. The coding scheme was reviewed by the first and second authors as a validity check. All participants' answers were then coded as having each of these themes present or absent. Approximately 20% of all responses were independently coded by the first author as a reliability check with almost complete agreement; any scoring differences, albeit negligible, were resolved through discussion.

### Statistical Analysis

All data were checked for accuracy and invalid data (e.g., any responses that were not plausible) were removed. Statistical analysis was performed using IBM SPSS version 26 (SPSS Inc., Chicago, IL). Descriptive analysis was performed to examine the characteristics of the sample and the distribution of the quality of care related outcomes. Means and standard deviations were calculated for continuous variables while proportions were calculated for categorical variables. An independent samples *t*-test was performed to compare QPCQ subscales reported by pregnant and postpartum women during the COVID-19 pandemic. Pearson correlations were used to support the bivariate analysis, which aimed to verify the association between the independent variables (maternal and obstetric care characteristics as well as COVID-19 related outcomes) and the dependent variable (quality of care during the gestational or post-partum period, i.e., QPCQ percentage score). Multiple linear regression analysis was then used to determine which of the analyzed variables could be considered predictors of maternal quality of care during a pandemic where QPCQ percentage score was the dependent variable. No statistical analysis was performed on the qualitative responses; these were collated for observational purposes only.

## Results

### Participant Characteristics

Of the 1,147 responses, 225 were removed due to incomplete consent (*n* = 125), ineligibility with regards to pregnant and post-partum status (*n* = 15) and/or no data being provided beyond consent (*n* = 90). Of the 917 eligible women, 612 (67%) were pregnant and 305 (33%) were postpartum. The mean age of participants was 31 ± 5.2 years (*n* = 911), 70% (*n* = 458) had at least 1 child already and 39% (*n* = 355) of the sample lived in cities. The majority of responses came from women educated beyond high school (*n* = 789), living in the United Kingdom (*n* = 625), of white ethnicity (*n* = 873), in a relationship (*n* = 861) and in full-time employment (*n* = 488; Table 1). Eighty-six percent (*n* = 796) of the sample rated their general health positively; 17% (*n* = 156) reported having ≥1 pre-existing health condition while 22% (*n* = 201) reported ≥1 pregnancy related complication. The prevalence of pre-existing and pregnancy related complications are illustrated in Table 1.

**Table 1:** Participant characteristics.

**Number (% out of 917) or** **±** **standard deviation**	
**Degree**	
Less than high school degree	6 (1%)
High school degree or equivalent	84 (9%)
College degree	182 (20%)
Bachelor degree	251 (27%)
Graduate degree	149 (16%)
Postgrad	207 (23%)
Other	25 (3%)
Prefer not to say	11 (1%)
**Ethnic background**	
White	873 (95%)
Black or African American	3 (0.3%)
American Indian	1 (0.1%)
Asian	17 (2%)
Native Hawaiian or Pacific Islander	21 (2%)
**Country of residence**	
Australia	12 (1%)
Bermuda	1 (0.1%)
Canada	25 (3%)
Germany	1 (0.1%)
India	1 (0.1%)
Indonesia	1 (0.1%)
Ireland	66 (7%)
New Zealand	2 (0.2%)
Pakistan	1 (0.1%)
UAE	5 (1%)
UK	652 (71%)
USA	129 (14%)
**Relationship status**	
Single	18 (2)
In a relationship/married, living together	861 (94%)
In a relationship/married, living apart	34 (4%)
Separated	1 (0.1%)
Widowed	2 (0.2%)
**Employment status**	
Student	17 (2%)
Self-employed	49 (5%)
Employed part-time	137 (15%)
Employed full time	488 (53%)
Homemaker/full-time parent	61 (7%)
Unemployed before COVID-19 and looking for work	5 (1%)
Unemployed before COVID-19 and not looking for work	10 (1%)
Employed before COVID-19 but have been laid off work during the pandemic	20 (2%)
I have been furloughed	100 (11%)
Other	29 (3%)
**Pre-existing complications**	
Cardiovascular disease	7 (1%)
Respiratory disease	35 (4%)
Type 1 diabetes	1 (0.1%)
Type 2 diabetes	2 (0.2%)
Impaired glucose tolerance	6 (1%)
High blood pressure	16 (2%)
Neurological disorder	10 (1%)
Depression	175 (20%)
Anxiety	219 (24%)
Bone disease	4 (1%)
Other	60 (7%)
Average number of complications pre-pregnancy	0.58 ± 0.88
**Pregnancy complications**	
No complications	637 (70%)
Gestational diabetes	51 (6%)
Preeclampsia	27 (3%)
Eclampsia	1 (0.1%)
Placenta previa	22 (2.4%)
Pre-term labor	13 (1.4%)
Intrauterine growth restriction	12 (1.3%)
Twins	18 (2.0%)
Short cervix	6 (1%)
Pelvic girdle pain	108 (12%)
Depression	31 (3%)
Anxiety	63 (7%)
Bone disease	1 (0.1%)
Other	43 (5%)
Average number of pregnancy related complications	0.43 ± 0.78

Five percent (*n* = 44) of the sample had experienced COVID-19 symptoms, 9% (*n* = 78) had received a COVID-19 test and <1% (*n* = 5) were diagnosed with a positive result. Forty-nine percent (*n* = 445) of the women had self-isolated of which 422 women clarified that the reason for this was due to medical reasoning (11%; *n* = 46), personal choice (62%; *n* = 260) and a combination of both (27%; *n* = 116). Of the entire cohort, 46% (*n* = 419) perceived themselves to be at higher risk in general because of COVD-19 compared to individuals who were not pregnant or in the first 6 months after delivery.

### Access to Healthcare

Fifteen percent (*n* = 141) of women indicated that COVID-19 had impacted how they traveled to appointments. During the pandemic, sixty-eight percent (*n* = 624) of women report traveling to pregnancy related clinical appointments by car. Forty-eight percent (*n* = 439) of women reported receiving care from a midwife, 16% (*n* = 143) from an obstetrician, 0.5% (*n* = 5) from a family doctor and 23% (*n* = 210) from a combination of services. Twenty-nine percent (*n* = 265) of pregnancy related appointments took place at a general practice, 33% (*n* = 302) at hospital, 8% (*n* = 77) at private clinics while 19% (*n* = 176) indicated *other* on the questionnaire (Table 2).

**Table 2:** Type and access to obstetric care during the COVID-19 pandemic.

**Type of healthcare provider**	**Number (% out of 917)**
Midwife	439 (48%)
Obstetrician	143 (16%)
Family doctor	5 (0.5%)
Combination of services	210 (23%)
**Place of healthcare**	
General practice	265 (29%)
Hospital	302 (33%)
Private clinic	77 (8%)
Other	176 (19%)
**Impact of COVID-19 on obstetric services**	
COVID-19 affected healthcare	571 (62%)
At least one appointment rearranged	166 (29%)^a^
At least one appointment canceled	167 (29%)^a^
Appointments fulfilled *via* or virtually means	175 (31%)^a^
Appointment cancellations on behalf of clinic	372 (41%)
Appointment cancellations on behalf of patient	81 (9%)
Scheduled appointments had taken place	639 (70%)
*(albeit type may be different)*	
Pregnancy services suspended	223 (36%)^b^
**Pregnant and postpartum women prefer to:**	
Physically attend appointments	676 (74%)
Have phone appointment	120 (13%)
Have a virtual appointment	85 (10%)
Have a home visit	77 (8%)
Miss appointments	16 (2%)

a *Subgroup of n = 571*;

b *Pregnant cohort only*.

Nine percent (*n* = 82) of women reported changing their healthcare provider because of COVID-19. Sixty-two percent (*n* = 571) of women reported that COVID-19 had affected their healthcare. Of this 571, 29% (*n* = 166) reported that at least one appointment had been rearranged, that at least one appointment had been canceled (29%; *n* = 167), 31% (*n* = 175) of women had their appointment over the phone or *via* virtual means while 10% (*n* = 57) reported *other* although did not disclose how their care was affected. Appointment cancellations/rescheduling on behalf of the clinic occurred for 41% (*n* = 372) of pregnant and postpartum women while 9% (*n* = 81) of women canceled appointments themselves predominantly due to childcare issues (*n* = 34) and concerns around availability of personal protective equipment (*n* = 28). Of the pregnant cohort, 36% (*n* = 223) reported that pregnancy-related services had been suspended because of COVID-19 that may have included, although not limited to, blood pressure, blood, and urine tests. The suspension of these services caused women to feel anxious (*n* = 84), neglected (*n* = 46), neutral (*n* = 39), sad (*n* = 31), and frustrated (*n* = 24) about this (Table 3). Seventy-four percent (*n* = 676) of respondents prefer to physically attend clinical appointments, 13% (*n* = 120) would prefer a phone appointment, 10% (*n* = 85) would prefer a virtual appointment, 8% (*n* = 77) would prefer a home visit while only 2% (*n* = 16) would prefer to miss their appointment during this time. These findings illustrate that the majority of women did not want to reduce the number of medical appointments, but due to clinic factors or their own personal circumstances, the number of appointments had reduced for around a third of women.

**Table 3:** Example responses when participants were asked how they felt about services being suspended/canceled.

**Feeling**	**Example quotes**
Anxiety	“As this is my first baby, I am extremely anxious. I am not from the U.K. (my husband is) and definitely feel that my care would have been better elsewhere” (P46). “Nervous—lack of reassurance & information” (P93). “Anxious and angry” (P225).
Neutral	“Ok, not ideal but the best of a difficult decision I think” (P590). “Feeling perfectly fine so have no concerns” (P919). “It reduces my contact with other people so it's preferable at this stage” (P302).
Sad	“Sad. This is my first baby, and I don't feel I'm getting the same experience regarding antenatal care as would be usual in normal circumstances” (P314). “Upset as haven't heard my baby's heartbeat at all” (P373). “Very unhappy about the safety my pregnancy especially after previous delivery problems” (P428).
Frustrated	“Frustrated and nervous that important appointments will be taken away from me. If grocery stores are deemed essential, how is it that many Dr. appointments get canceled? They are essential services!” (P459). “Frustrated and disappointed. I have had no support or contact from the community midwife team. My hypertension diagnosis was almost missed due to lack of appointments” (P666). “It made me very frustrated. I understood why but I felt like I wasn't able to make fully informed decisions or know if I would need further testing or not” (P445).
Neglected	“A bit abandoned as first pregnancy and would like confirmation that all is ok” (P87). “Neglected. Not getting the care I was originally told I needed” (P214). “I have never actually spoken nor met my assigned midwife, for all appointments I have had to chase or ascertain whether they were going ahead, and *via* which means” (P952).

### Access to Birthing and Postpartum Services

Of the pregnant cohort, 72% (*n* = 441) planned to have a vaginal birth with 83% (*n* = 507) reporting that COVID-19 had not influenced delivery mode. Pregnant women mostly planned to deliver in hospital (77%; *n* = 470) with 10% reporting that the COVID-19 pandemic had altered plans about where they would deliver. Only pregnant women were asked about their birth partner; 85% (*n* = 518) confirmed they had a birth partner and 77% (*n* = 471) reported that having birth partners present at delivery was viewed as important. Four percent (*n* = 22) of pregnant women were permitted accompaniment of birth partners to clinical appointments, 62% (*n* = 380) were allowed have their birth partner at delivery, 18% (*n* = 111) were unsure of the rules regarding partner attendance, while 4% (*n* = 26) were not permitted to attend delivery. For pregnant women unsure about their birth partners permissions or not permitted accompaniment (*n* = 137), feelings of anxiety (65%; *n* = 89), sadness (39%; *n* = 54), and loneliness (9%; *n* = 12) were reported while those permitted to have birth partners present expressed feelings of relief (33%; *n* = 124) (Table 4). A total of 66% (*n* = 401) women planned to breastfeed, with 47% (*n* = 289) expecting adequate support. Forty-nine percent (*n* = 300) pregnant women expected to receive a home visit by a community midwife. Of the postpartum cohort, 50% (*n* = 150) chose to breastfeed and 21% (*n* = 64) of respondents reported they did not receive adequate help. Forty-nine percent (*n* = 149) of post-partum women received a community visit postpartum with a negligible number of women uncomfortable with this (1%; *n* = 4).

**Table 4:** Example responses when participants were asked how they felt about their partners restricted access or permission to attend delivery.

**Feeling**	**Example quotes**
Anxiety	“He's only allowed in once I'm 7 cm dilated & I'm worried about that. I want him there from the start as I think I'll panic without him” (P93). “I'm being induced, and he can't attend until I am in labor so very scared and worried as it is my first baby” (P241). “Nervous and worried about not having support with me” (P315).
Relieved	“Reassured. I know I will need the support” (P34). “Yes, thank God, he can be there. I am so relieved I won't be alone and that he can share this huge transition in our lives with me” (P66). “I am very glad I will be allowed one support person in the delivery room” (P63). “I'm glad he is able to be with me as I do not want to be on my own and do not want my husband to miss the birth of our first child” (P104).
Sad	“This will probably be our only baby and that he hasn't been able to come to my scans has been very disappointing and upsetting” (P291). “Upset as I wish to have him there throughout the whole time” (P373). “Upset and angry” (P649).
Loneliness	“They can only be present for the very end stage of labor and cannot visit afterwards. I feel alone, extremely anxious and devastated” (P9). “It makes me feel scared that I will have to go through such a beautiful and scary time all alone especially it being a first child” (P412) “He won't be allowed to join until 3 cm—this makes me feel lonely and isolated” (P786).

### Quality of Care

The subscales and overall scores of the QPCQ are displayed in Table 5. Of the entire cohort, 620 completed the QPCQ of which *n* = 453 were pregnant while *n* = 167 were postpartum. Of those that completed the QPCQ, 66% (*n* = 297) of pregnant and 75% (*n* = 126) of postpartum respondents perceived their quality of care as “*good”* (>70%), while 34% (*n* = 156) of pregnant women and 25% (*n* = 41) of the post-partum women reported their quality of care as “*poor”* (<70%). This equates to overall, 68% of women reporting good quality of care with 32% reporting poor quality of care. Postpartum women scored significantly higher on approachability (*p* = 0.04), anticipatory guidance, sufficiency, and support compared to pregnant women (*p* < 0.001; Table 5).

**Table 5:** Quality of Prenatal Care Questionnaire subscale and total scores for the entire sample, pregnant and postpartum cohort.

**Subscale**	**Entire group**	**Pregnancy**	**Postpartum**	***p*-value**
Information sharing	4.1 ± 0.7	4.1 ± 0.7	4.2 ± 0.8	0.13
Anticipatory guidance	3.4 ± 0.9	3.3 ± 0.8	3.6 ± 0.9	<0.001
Sufficiency	3.6 ± 0.9	3.5 ± 0.9	3.7 ± 0.9	<0.001
Approachability	3.8 ± 0.9	3.8 ± 0.9	4.0 ± 0.8	0.04
Availability	3.7 ± 0.9	3.7 ± 0.8	3.8 ± 0.9	0.42
Support	4.0 ± 0.8	3.9 ± 0.7	4.1 ± 0.8	<0.001
Total Score (out of 5)	3.7 ± 0.8	3.7 ± 0.7	3.7 ± 0.8	0.24
Total Score (% of maximum)	74 ± 16	74 ± 14	76 ± 19	0.05

A number of factors within the pregnant cohort were significantly correlated with the QPCQ percentage score including the country within which participants lived (*r* = −0.95, *p* = 0.05). To allude further, QPCQ score differed for those living in Australia (83 ± 12%; *n* = 8), Canada (80 ± 14%; *n* = 14), Ireland (72 ± 10%; *n* = 23), UAE (72 ± 15%; *n* = 4), UK (72 ± 14%; *n* = 314), and USA (77 ± 15%; *n* =73). Self-rated health was significantly correlated with QPCQ score (*r* = 0.14, *p* = 0.002), with positively rated health favoring good quality of care.

Respondents that received obstetrician and midwife care yielded perceived quality of care to be favorable (*r* = −0.11, *p* = 0.02) over those receiving care from a family doctor or a combination of services. Service cancellations (*r* = 0.23, *p* < 0.001), suspension to services (*r* = 0.18, *p* < 0001) and changes made to planning delivery mode (*r* = 0.15, *p* = 0.001) were all significantly correlated to QPCQ score whereby those that were negatively impacted, reported lower quality of care. Quality of care was significantly correlated with birth partners permission to attend birth (*r* = −0.21, *p* < 0.001) whereby those permitted to be accompanied reporting good quality of care (76 ± 14%) compared to those not permitted (63 ± 10%) and those that were unsure (69 ± 14%). In contrast, birth partner attendance to clinical appointments was not associated with quality of care (*r* = −0.06, *p* = 0.19).

These outcomes collectively generated a significant multiple linear regression model, with QPCQ score as the dependent variable; *F*_(7, 433)_ = 11.5, *p* < 0.001, *R*^2^ = 0.16 (Table 6). Of these factors, birth partners attendance and appointment cancellations significantly contributed to the QPCQ score (*p* < 0.001) as did self-rated health, type of healthcare received, suspension to services, and changes to delivery mode (*p* < 0.05). For postpartum women, the only factor associated with the QPCQ result was the support available to breastfeed (*r* = 0.28, *p* = 0.001) which contributed significantly to the overall QPCQ score according to linear regression analysis; *F*_(1, 147)_ = 12.05, *p* = 0.001, *R*^2^ = 0.08 (Table 6).

**Table 6:** Multiple Regression Predicting Quality of Care for pregnant and postpartum women.

	**β (95% CI)**	***p*-value**
**During pregnancy**		
Country residing	−0.08 (−0.6, 0.01)	0.09
Self-rated general health	0.11 (0.4, 3.5)	0.01
Type of healthcare	−0.09 (−2.4, −0.1)	0.04
Clinic canceling or rescheduling	0.18 (2.5, 7.7)	<0.001
Services suspended	0.12 (0.6, 4.6)	0.01
Delivery mode influenced by COVID-19	0.13 (1.7, 9.2)	0.004
Birth partner attending delivery	−0.21 (−5.1, −2.0)	<0.001
**Post-partum**		
Adequate help breastfeeding	0.28 (4.63, 16.9)	0.001

### Future Information

A subset of pregnant women (*n* = 296) responded to an open question asking what information would be useful for them to have during a time where physical distancing is in place. Of this subset, respondents indicated that it would be beneficial to receive information on the risks associated with COVID-19 for mother and baby (17%; *n* = 50), remain updated on changes made to services pertaining to scheduling (what appointments to expect next), cancellations, and clarity on rules for birth partners to attend routine appointments including delivery (28%; *n* = 84). Women would benefit from guidance on delivery options including pain relief, induction, birth plans, and home births (28%; *n* = 78), antenatal classes available to meet other pregnant women (7%; *n* = 21), breastfeeding (5%; *n* = 16), and mental health (2%; *n* = 46). The majority of women reported that they engaged with Facebook for pregnancy related information (88%; *n* = 260) with Instagram (18%; *n* = 53) and Mumsnet (5%; *n* = 14) also used. The preferred form of information varied between an infographic (35%; *n* = 105), leaflet (30%; *n* = 88), video (29%; *n* = 85), and an online Q&A (19%; *n* = 55).

A subset of post-partum women (*n* = 155) responded to an open question asking how post-partum care could be improved or altered during a time where physical distancing is in place. Twelve percent (*n* = 18) of women stated no improvement in care was necessary, 40% (*n* = 62) proposed virtual appointments would be useful while 15% (*n* = 24) indicated that more PPE to allow for face-to-face appointments would be beneficial. Lastly, 29% (*n* = 45) said they would benefit from more support that could be achieved with less rushing and canceling of appointments.

The same subset indicated that recently pregnant women would benefit from more information regarding risks related to COVID-19 and advice on how to stay safe during pregnancy (21%; *n* = 33), more general pregnancy related information (including antenatal and postnatal classes, labor, delivery and any reference to support that is available) (27%; *n* = 42), updates regarding any changes to services as a result of COVID-19 (27%; *n* = 42), and advice on how to cope with loneliness following birth particularly in the absence of peer contact that has been removed because of the pandemic (17%; *n* = 27). Postpartum women predominantly engaged with Facebook (12%; *n* = 35) for pregnancy related information while Instagram and Mumsnet were preferred for others (6%; *n* = 19 and 2%; *n* = 6, respectively).

## Discussion

This study demonstrated that patient-reported perceptions of obstetric health care quality was negatively impacted by disturbances to services (cancellations and suspensions) and ambiguity regarding birth partner permissions to attend delivery during the COVID-19 pandemic. The majority of women indicated a willingness to continuing to attend appointments in person. Finally, women identified a need for clearer communication predominantly regarding any changes to maternity services (scheduling, cancellations, and services available), clear guidance on birth partner permissions to attend routine appointments including delivery and clarity regarding the associated risks of COVID-19 for mother and baby.

### Access to Healthcare

In this study, the majority of scheduled pregnancy related appointments were fulfilled, however, more than one-third of women experienced suspension to services and consequentially, anxiety, frustration, and sadness (Table 2). In support of fulfilling services and avoiding suspensions and cancellations, healthcare providers can benefit in knowing that although half of the respondents in this study felt at higher risk because of COVID-19 compared to a non-pregnant or non-post-partum woman, most wanted to physically attend appointments. The suspension of services may be due to inadequate personal protective equipment (PPE) supplies (28) to protect women and healthcare staff against contracting COVID-19 (29, 30). In situations where this is not feasible, alternative strategies could be adopted to alleviate associated stress and anxiety for women and may include virtual appointments, phone calls or where feasible, a home visit. These strategies, that appear to be accepted by many, offer greater flexibility for healthcare professionals to ensure services can be maintained in an effort to avoid suspensions and cancellations to obstetric care.

Birth partners have been heavily impacted by the COVID-19 pandemic with many excluded from or unclear about their permissions to attend appointments and including delivery. According to respondents, it is important to women to have the accompaniment of their birth partner at delivery (Table 4). It is noteworthy that high quality of care was associated with having a birth partner at delivery, although not associated with accompaniment to regular clinical appointments. This interesting observation highlights the importance of prioritizing birth partner attendance at delivery over all other appointments which could potentially alleviate the anxiety and loneliness for those giving birth during the COVID-19 pandemic. The rules vary greatly between countries (31); in the United States, many clinical settings are excluding birth partners or requesting a choice between a doula and birth partner (4), while in the UK, asymptomatic birth partners are permitted to be present for labor and birth whilst wearing a face mask, unless performed under general anesthetic (2). Indeed, such precautions are in place to minimize the risk of COVID-19 infection to the mother and infant. However, support from partners and caregivers have many positive effects on maternal health and well-being at delivery (4) including reduced labor pain, reduced stress, shorter duration of labor, less medication need, increased maternal satisfaction, and a positive attitude toward motherhood (32, 33). Furthermore, emotional support has shown to reduce the length of stay in hospital and the need for delivery by cesarean section (34). Therefore, the absence of maternal support in an effort to protect against COVID-19 contamination may adversely affect other aspects of maternal and childbirth outcomes that could have long lasting implications. Birth partners are also helpful for hospital staff who have reported that they feel bad when they are unable to provide one-to-one support during the pandemic in the absence of birth partners (4). This is reaffirmed by one respondent in this study who stated that “I feel it is important for my partner to be there for his baby's birth and feel that pressure will be taken off of midwives if a partner is there to assist” (P955). Based on the positive outcomes that birth partner attendance can have on maternal and infant outcomes as well as alleviating pressures for hospital staff, facilitating birth partner attendance to delivery seems imperative.

### Quality of Care

Disruption to obstetric services including suspensions, cancellations, changes to delivery plans, restricted birth partner access, and inadequate breastfeeding support were associated with reduced quality of care during pregnancy and the postpartum period. Despite this, the overall influence of these factors in collectively predicting quality of care was low. This may in part be explained by the understanding that quality of care is a multi-dimensional concept and includes a variety of characteristics including safety, efficacy, timely, efficiency, equity, and a people-centered approach to care (35). While this study primarily focused on the timely aspect of care (reducing delays in providing/receiving healthcare), it is plausible that other factors, for example, safety (delivering healthcare that minimizes risks and harm to service users), may have impacted quality of care to a greater extent particularly during the COVID-19 pandemic. In agreement with a previous study, our findings highlight that post-partum women would benefit from increased breastfeeding support (36). This may be of unique value to healthcare professionals to ensure bonding between mother and baby particularly at a time where anxieties and psychological vulnerability are heightened (37–39).

### Future Information

This study has provided insight into what pregnant and postpartum women would like to know during a pandemic. Pregnant women want to be informed about the logistics of having a baby during a pandemic, including site specific changes to services and rules regarding birth partners permission to attend delivery. As aforementioned, these factors were associated with quality of care; it is plausible that if women received sufficient information about service changes and birth partner permissions, the psychological burden of COVID-19 on pregnancy related care could be lessened. Virtual appointments seemed acceptable by postpartum women and the logistics of this have previously been described (40). Postpartum women also want to receive guidance on reducing loneliness that appears to be a common feeling for this population during the COVID-19 pandemic (41). This warranted information could be shared *via* social media platforms with no clear preference identified by participants regarding the format of delivery.

### Limitations and Future Directions

This study is limited by a reliance on participants to self-report their eligibility (pregnant or within 6 months after delivery) to participate. Secondly, although this survey was distributed with intention of reaching a global audience, the majority of respondents were white and from developed countries including the United Kingdom, Ireland, and the United States. This limits the generalizability of findings to all pregnant and/or recently post-partum women. Lastly, to the authors knowledge, no valid questionnaire exists to quantify access of healthcare specifically among pregnant and postpartum women. Although all constructs of healthcare access have been described, it may limit the replicability of this outcome.

This study converges with previous authors whereby healthcare services become disrupted during a pandemic and that these disruptions are negatively associated with quality of care (17, 18). The research has also provided agreement that women feel anxious during this time (42) and that this is likely exacerbated by birth partner restrictions (4). To the authors knowledge, this is the first study during the COVID-19 pandemic to attempt to quantify access to and quality of obstetric care. Lastly, this study offers novel insight into the information and guidance wanted by pregnant and post-partum women during this time. While suggestions were made by Jago et al. (13) regarding what this information could be, this study presents primary data to support their suggestions. Given that this [information sharing] is a construct of prenatal quality of care (19) and can be achieved by an online distribution of resources, it is an important confirmation from respondents that could ease in part, the burden of the COVID-19 pandemic on pregnant and postpartum women. To advance on this work further, insight is necessary to understand access to, and quality of care for pregnant and post-partum women from the black and ethnic minority community and from countries not captured in this study.

## Conclusion

During this global pandemic, many pregnant and postpartum many women have experienced a disruption in their access to healthcare. Patient perceptions of the quality of their obstetric care was negatively influenced by cancellation of appointment(s), suspension of services and exclusion of birth partners at delivery. Accordingly, ensuring the continuity of care *via* virtual and/or phone appointments and providing clear guidance on birth partner permissions to attend delivery may help improve quality of obstetric care during this time.

## Data Availability Statement

The raw data supporting the conclusions of this article will be made available by the authors, without undue reservation.

## Ethics Statement

The studies involving human participants were reviewed and approved by Ethics Committee at York St. John University (Reference number: STHEC0011). The patients/participants provided their written informed consent to participate in this study.

## Author Contributions

AB had access to the data in the study and takes responsibility for the integrity, the accuracy of data, and statistical analysis. FL data analysis. HJ and MD study concept and design. All authors data interpretation, drafting, and revisions of manuscript for important intellectual content.

## Conflict of Interest

The authors declare that the research was conducted in the absence of any commercial or financial relationships that could be construed as a potential conflict of interest.
